# Advantages of analysing both pairwise SNV-distance and differing SNVs between *Mycobacterium tuberculosis* isolates for recurrent tuberculosis cause determination

**DOI:** 10.1099/mgen.0.000956

**Published:** 2023-03-23

**Authors:** Darja Sadovska, Anda Nodieva, Ilva Pole, Jānis Ķimsis, Anda Vīksna, Iveta Ozere, Inga Norvaiša, Dace Bandere, Renāte Ranka

**Affiliations:** ^1^​ Latvian Biomedical Research and Study Centre, Riga, Latvia; ^2^​ Riga East Clinical University Hospital, Centre of Tuberculosis and Lung Diseases, Stopiņi Region, Upeslejas, Latvia; ^3^​ Department of Infectology, Rīga Stradiņš University, Riga, Latvia; ^4^​ Department of Pharmaceutical Chemistry, Rīga Stradiņš University, Riga, Latvia

**Keywords:** recurrence, reactivation, reinfection, tuberculosis, whole genome sequencing

## Abstract

Endogenous reactivation and exogenous reinfection are two possible causes of recurrent tuberculosis (TB). However, in some cases, precise cause determination can be challenging. In this study, we used whole genome sequencing to determine pairwise SNV distances and detect differing SNVs in initial and subsequent isolates for recurrent TB cases when the first and second episodes were caused by *Mycobacterium tuberculosis* (*Mtb*) strains with an identical spoligotype pattern. In total, 104 *Mtb* isolates from 36 recurrent TB and 16 single TB episode patients were included in the study. Most isolate pairs belonged to the SIT1 (n=21), SIT42 (n=9), SIT53 (n=9), and SIT254 (n=7) spoligotypes, and in 27 cases, resistance to at least one anti-TB drug was found in either isolate. Drug susceptibility was more common in the recurrent TB patient cohort, and longitudinal single TB episode isolates were more prone to be drug-resistant (p=0.03), while the association between patient cohort and spoligotype was not statistically significant (p=0.07). The pairwise SNV-distance between the longitudinal single TB episode isolates was small (0-7 SNVs). Among the recurrent TB isolates, based on the high SNV-distance (38–273 SNVs), six reinfection cases (16.7%) were identified. This distance was small (<10 SNVs) in the remaining 30 isolate pairs. Further analysis of differing SNVs revealed that 22 (61.1%) cases could be classified as possible reactivation. Notably, despite the small distance of 2–7 SNVs, initial isolates of eight patients (22.2%) had several SNVs that were not found in the second isolates; therefore, these cases were classified as reinfection with a closely related *Mtb* strain. No statistically significant difference in the time interval between specimen collection in the reactivation and reinfection *Mtb* sample groups (p=0.13) or an association between recurrence cause and drug resistance status (p=0.62) or spoligotype (p=0.79) could be detected. The mycobacterial median mutation rate of longitudinal single TB episodes and possible reactivation isolate pairs (n=37) was 0.12 SNVs/genome/year (IQR 0-0.39), and in 18 cases (48.6%), it was equal to zero. No statistically significant differences in mutation rate were found between recurrent TB and longitudinal single TB episode isolates (p=0.087), drug-susceptible and resistant isolates (p=0.37) or isolates of Beijing and other genotype families (p=0.33). Furthermore, four cases of fluoroquinolone resistance development through the acquired SNVs in the *gyrA* gene were identified. To conclude, this study highlighted the complexity of recurrent episode cause determination and showed the usefulness of differing SNV identification in both *Mtb* isolates in such cases. Expected drug susceptibility was the only discriminative factor for recurrent TB episode-causing mycobacterial strains, while no differences between reactivation and reinfection sample groups could be identified.

## Data Summary

The raw sequencing reads obtained in the study were deposited in European Nucleotide Archive (ENA) under the project accession number PRJEB53131.

**Impact Statement**

Recurrent active *Mycobacterium tuberculosis* infection is a significant obstacle for efficient tuberculosis control worldwide. Effective prevention measures can only be implemented when a deciphering algorithm between two possible causes of recurrent episodes (endogenous reactivation and exogenous reinfection) is accurate, standardised and well understood. This work complements a previously suggested approach of pairwise SNV-distance determination using whole genome sequencing data by adding the detection of differing SNVs in initial and subsequent *M. tuberculosis* isolates, which made the identification of reinfection cases with closely related mycobacterial strains possible. Moreover, to obtain a more detailed description of tuberculosis recurrence causing *M. tuberculosis* strains, different mycobacteria-associated factors were compared between possible reactivation and reinfection cases, as well as between all recurrent tuberculosis patients and a cohort of single tuberculosis episode experienced patients. Furthermore, we report four cases of additional drug resistance development through an acquired SNV.

## Introduction

Tuberculosis (TB) is an infectious disease caused by the bacillus *Mycobacterium tuberculosis* (*Mtb*). It is still a major health threat worldwide, and before the onset of the COVID-19 pandemic, it was a leading cause of death from a single infectious agent [1]. One of the goals of the World Health Organization’s (WHO) endorsed End TB Strategy is reducing the TB incidence rate, which comprises both new and recurrent cases [2], and highlights a recurrent active infection as an important obstacle for successful disease control.

Recurrent TB is defined as the repeat occurrence of an active TB disease after successful treatment of the primary episode, i.e., when the outcome can be classified as “cured” according to WHO guidelines [3]. There are two possible causes of recurrent TB: endogenous reactivation of previously acquired *Mtb* infection, also referred to as relapse, or exogenous reinfection with distinct mycobacterial strains [4]. To introduce efficient TB control measures and identify high-risk groups of previously cured patients, precise cause determination of recurrent TB episodes is crucial.

Initially, conventional genotyping methods, such as spoligotyping, insertion sequence *6110* restriction fragment length polymorphism (RFLP), or mycobacterial interspersed repetitive unit-variable number of tandem repeat (MIRU-VNTR) analyses, were used for this purpose [5–8]. If genotypes of initial and subsequent *Mtb* isolates matched, endogenous reactivation was thought to be a cause, and if genotypes were different, reinfection was a conclusion. However, this approach could not identify reinfection cases involving the same *Mtb* genotype. Whole genome sequencing (WGS) has greater discriminative power than previously mentioned genotyping methods, and it has been commonly used in *Mtb* drug resistance prediction, outbreak investigation, and strain phylogeny studies [9]. In recurrent TB cause investigation studies, WGS was simultaneously used with other genotyping methods. Afterward, the distinguishing algorithm based on SNV-distance cutoffs between isolate pairs was proposed [10–14]: in the case of endogenous reactivation, a small pairwise SNV-distance of ≤5 (up to 10–12) SNVs is expected, whereas a distance of ≥100 SNVs would indicate reinfection with a different strain. However, these thresholds were mostly determined in low-burden settings and could be arbitrarily interpreted. The threshold for endogenous reactivation is also widely accepted as an indicator of a recent transmission event [15–17].

Although these suggested SNV distances mostly corresponded with the results of different genotyping methods, inconclusive recurrent TB cases were also reported when SNV distances were between the defined thresholds (<100 SNVs), and these cases were classified as reinfections [11, 18]. Moreover, recently conducted studies showed that deciphering the cause of recurrent active TB infection is more complicated if a subsequent episode develops during an outbreak [19–21]. In this case, pairwise SNV-distance was insufficient to identify reinfection with a closely related strain. It needed to be complemented with the delineation of transmission roots within the outbreak and epidemiological data of involved patients. At this point, the algorithm of precise differentiation between reactivation and reinfection, if initial and subsequent episodes were caused by the same *Mtb* genotype, is still relevant.

Latvia is a low-moderate TB incidence country and one of the WHO priority countries in the European region [22]. The overall TB incidence was 23 new and relapsed cases per 100 000 in 2020 [23]. Diverse *Mtb* genotypes of Lineages 2 and 4 were frequently isolated from Latvian TB patients [24], while *Mtb* drug resistance and widespread multidrug- (MDR) and preextensively drug-resistant (pre-XDR) strains have been significant health care challenges for decades [25]. In this study, we used the WGS approach to identify pairwise SNV distances and detect differing SNVs between *Mtb* isolates obtained from pulmonary TB patients who experienced recurrent episodes of active infection caused by the same *Mtb* genotype as the initial episode. Simultaneously, various mycobacteria-associated factors (drug resistance, genotype, accumulated SNVs, molecular clock) were compared between recurrent TB isolates and longitudinal single TB episode isolates to identify recurrence-causing strain characteristics. In addition, potentially helpful discriminative factors between reactivation and reinfection (time between episodes, *Mtb* drug resistance status, and genotype) were assessed.

## Methods

### Sample collection

This retrospective study was conducted on pulmonary TB patients diagnosed in 2002-2019 in the Centre of Tuberculosis and Lung Diseases, Riga East Clinical University Hospital, the nationwide TB diagnostic, and treatment centre. According to the National Health Service register data, during the 2005-2017 period, the total number of TB cases in Latvia was 12484, including 1705 recurrent cases, and the estimated number of recurrent TB cases in the study period was 2500. For this study, all available paired *Mtb* isolates with identical spoligotypes that were acquired from TB patients with at least two-month intervals were included. Assigned patient and *Mtb* isolate identification codes are comprised in Supplementary material 1 (available in the online version of this article). Mycobacterial DNA samples were extracted from cultures grown on Löwenstein–Jensen media at the time of diagnosis according to the cetyltrimethyl ammonium bromide (CTAB) protocol [26] and were kindly provided by the clinical laboratory of the Centre of Tuberculosis and Lung Diseases. Spoligotyping was performed using commercially available reagent kits (Isogen Life Science, Netherlands; later – Ocimum Biosolutions, India) following a previously published standard protocol [27]. Spoligotype SIT numbers were inferred using the SITVIT2 database (available at http://www.pasteur-guadeloupe.fr:8081/SITVIT2/).

Hospital medical records and National Health Service register data were studied to compile the results of phenotypic drug susceptibility testing (pDST) and to divide *Mtb* samples into two groups – recurrent TB isolates and longitudinal single TB episode isolates. pDST was conducted at the Centre of Tuberculosis and Lung Diseases clinical laboratory according to up-to-date WHO guidelines, which were in effect at the time of the test performance. Only treatment outcome in either source defined as “cured” before the second isolate acquisition date was considered evidence of TB recurrence (first group). Bacteriologically confirmed pulmonary TB cases at the onset of treatment were classified as "cured" according to the WHO definition: patients, who completed the assigned treatment course according to the national policy, are clinically healthy with evidence of a bacteriological response and without any indications of treatment failure [3]. *Mtb* sample pairs acquired before bacteriological conversion, and isolates representing cases of patient noncompliance, incomplete treatment course, and treatment failure, made the second isolate group (i.e., longitudinal single TB episode isolates).

### WGS and bioinformatic data processing

Mycobacterial DNA samples were sheared physically using a Covaris S220 sonicator. Single-end fragment libraries were prepared using the Ion Plus Fragment Library Kit (Thermo Fisher Scientific, US) according to the manufacturer’s protocol. Samples were sequenced on an Ion Proton system (Thermo Fisher Scientific, US). Reads of maximum 200 base pairs were produced.

Bioinformatic sequence data analysis was performed using tools on the Galaxy web platform at https://usegalaxy.org public server [28], and the developed pipeline was based on the one proposed by the Galaxy community hub [29]. First, quality control of the obtained sequences was performed. Filter by quality (v1.0.2) was used to keep sequencing reads with Phred quality scores of at least 10 for 95 % of nucleotides and at least 20 for 80 % of nucleotides. An output was trimmed for adapter content, and low-quality ends (Phred quality score <20) using Trim Galore! (v0.6.3), keeping sequences longer than 30 base pairs. Low-quality reads (average Phred quality score <20) and reads of inappropriate length (longer than 200 bp) were then discarded by Filter FASTQ (v1.1.5).

Mapping of the output to the reference sequence and SNV calling, gene-based annotation, and functional effect prediction were performed using the snippy (v3.6.0) tool. The genome of the inferred *Mtb* complex’s most recent common ancestor, which was combined with the annotation of the H37Rv sequence (GenBank NC000962.3), was used as a reference [30]. Generated BAM file was used as an input for the TB-Profiler tool to determine WGS-based *Mtb* sublineage assignment. SNVs located in repetitive or insertion sequence sites, PE/PPE genes, or close to indels (within a five-nucleotide window) were discarded using TB Variant Filter (v0.3.5). Each detected variant had to be supported by at least four sequencing reads representing both forward and reverse strands with mapping quality scores greater than 20. Only homozygous variants (allele frequency ≥90%) were included in the analysis. Finally, to identify differing SNVs between each patient’s isolates, variant sites containing VCF files were merged using VCFcombine (v1.0.0), and all differences were checked manually using Integrative Genomics Viewer (IGV, v2.5.3). Samtools stats (v2.0.2) and plotCoverage (v3.3.2.0.0) were used to assess the average base quality of the mapped reads and reference genome coverage, respectively. Further analysis of gene-based annotation and functional effect prediction were performed on differing SNVs detected in isolate pairs where pairwise SNV-distance was ≤10 SNVs (recently acquired SNVs).

### Phylogenetic tree construction

Multiple sequence core alignment of the whole dataset was created using the snippy-core (v4.6.0) tool. The maximum likelihood method was used to estimate an outgroup-rooted phylogenetic tree with 500 bootstrap replications in MEGA11 software. The General Time Reversible (GTR) model with a proportion of invariant sites (GTR +I) was determined as the best-fit evolutionary model using the ModelTeller online tool (available at https://modelteller.tau.ac.il/). The phylogenetic tree was visualized and annotated on the iTOL server (v6; available at https://itol.embl.de/).

### Statistical analysis of acquired data

All calculations, statistical analysis, and data visualisation were performed in R (4.1.2) software. A level of significance α=0.05 was chosen for all tests. The distribution normality of quantitative data was checked using three approaches: construction of both quantile diagram and boxplot and performing Shapiro-Wilk test. If data were normally distributed, the mean value and standard deviation were used as descriptive statistics parameters; otherwise, the median and interquartile range (IQR) were reported. The chi-square test of independence, chi-square goodness of fit test, and Fisher’s exact test were performed on various category data. In contrast the Mann–Whitney–Wilcoxon test was used to analyse acquired quantitative data. Bayes factor upper bound and effect size with 95% confidence interval were calculated and reported for all performed nonparametric tests.

## Results

### Sequencing output quality, phylogeny, and dataset characteristics

WGS was performed on 104 mycobacterial isolates from 52 TB patients diagnosed in 2002–2019. According to the hospital medical records and National Health Service register data, 72 *Mtb* samples were obtained from recurrent TB patients, while 32 were classified as longitudinal single TB episode isolates (Table 1). For all sequenced *Mtb* DNA samples, the median base quality score was 26.1 (IQR 26–26.2), and the reference genome was covered with a median depth of 57.4 (IQR 50.1–77.2) reads per base. All isolates were kept for further data processing.

**Table 1.** Distribution of *Mtb* genotypes and drug resistance types in the studied isolate groups

Genotyping			Longitudinal single episode isolates (*n*=32)				Recurrent tuberculosis isolates (*n*=72)				Total (*n*=104)
L*	SL*	SIT (F)†	S (31.2%)	HR (6.3%)	MDR (34.4%)	pre-XDR (28.1%)	S (56.9%)	HR (16.7%)	MDR (19.4%)	pre-XDR (6.9%)	
2	2.2.1 (Beijing)	1 (Beijing)	4		5	5	9	6	9	4	42
		190 (Beijing)				2					2
4	4.1.2.1 (T1; H1)	47 (H1)					2				2
		283 (H1)		2							2
	4.2.1 (Ural)	1117 (Ural-1)							2		2
	4.3.3 (LAM)	42 (LAM9)			6	2	6		3	1	18
		254 (LAM-RUS)	2				12				14
	4.8 (mainly T)	40 (T4)	2								2
		53 (T1)	2				10	6			18
		156 (S)					2				2

*TB-Profiler genotyping results.

†Spoligotyping results.

F, family; HR, isoniazid-resistant; L, lineage; MDR, multidrug-resistant; pre-XDR, preextensively drug-resistant; S, drug-susceptible; SL, sublineage.

**Fig. 1.**
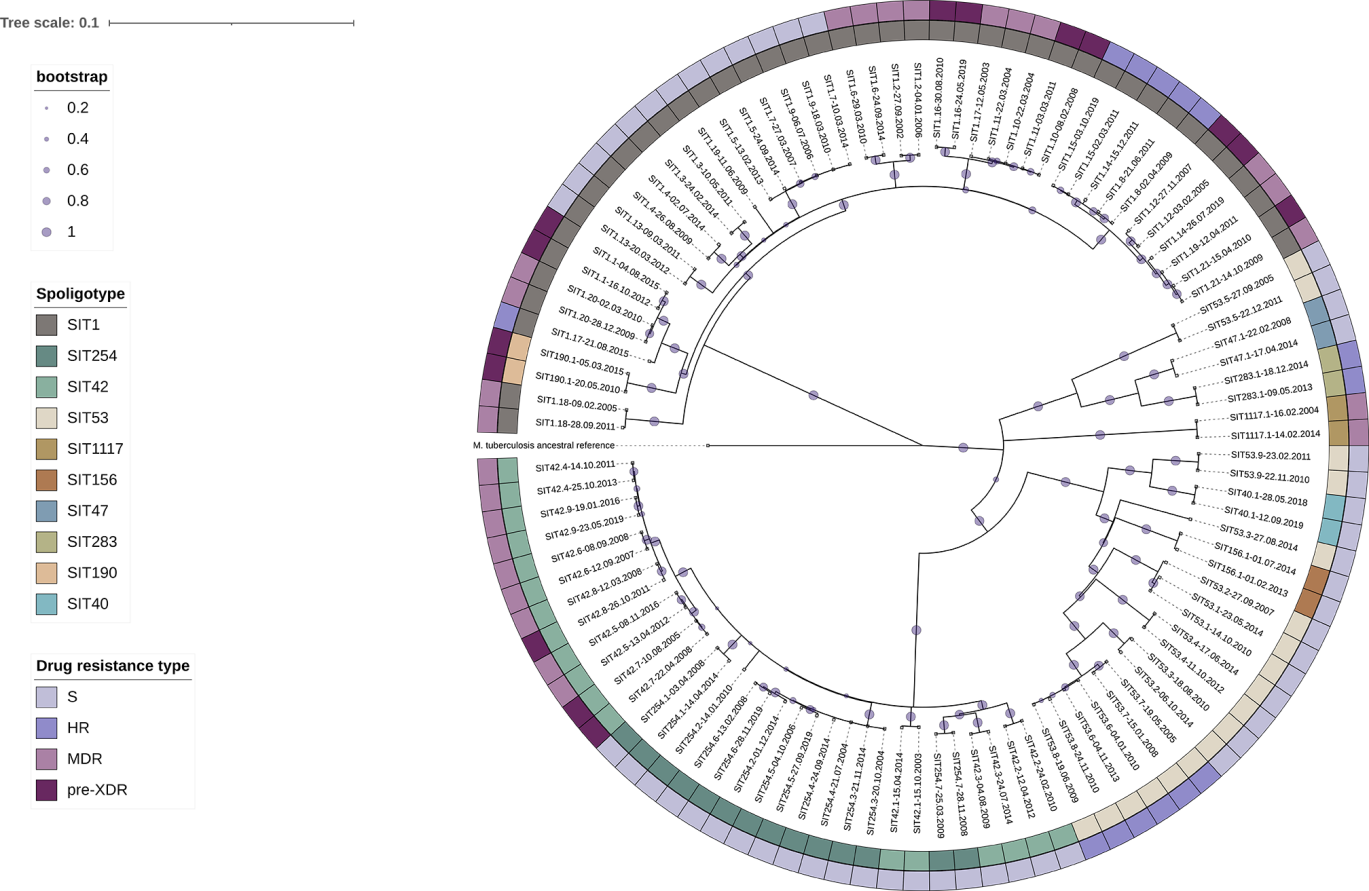
Maximum-likelihood phylogeny of the studied dataset. Abbreviations: S – drug-susceptible, HR – isoniazid-resistant, MDR – multidrug-resistant, pre-XDR – preextensively drug-resistant.

The spoligotyping results showed that *Mtb* isolates represented various genotypes (Fig. 1, Table 1). Most samples belonged to the Beijing (SIT1, SIT190) and LAM (SIT254, SIT42) families. Other isolates represented (in descending order) T (SIT53, SIT40), H (SIT283, SIT47), Ural (SIT1117), and S (SIT156). The TB-Profiler generated genotyping results and spoligotyping data were compared. Although *Mtb* genotype determination by both methods was identical, spoligotyping demonstrated greater resolution in this dataset. Therefore, for further *Mtb* sample characteristics and statistical analysis spoligotyping assigned genotypes were used.

The created phylogeny of the dataset shows that within the Beijing and LAM families, *Mtb* isolates demonstrated greater genetic relatedness than in other clades. Drug-resistant isolates formed several monophyletic groups, following similarities of resistance types in the SIT1, SIT42, and SIT53 genotypes.

*Mtb* samples pairs from most patients belonged to the SIT1 spoligotype (*n*=21), and several showed corresponding SIT42 (*n*=9), SIT53 (*n*=9) and SIT254 (*n*=7) patterns. Six other spoligotypes (SIT40, SIT47, SIT156, SIT190, SIT283, and SIT1117) were identified in one TB patient each, and for further statistical analysis, these samples were referred to as ‘rare’ spoligotypes.

In 25 patients, both *Mtb* isolates were drug susceptible. In 26 patients, both *Mtb* isolates were resistant to at least one medication, and in one more case (P46SIT1.19), only the second isolate showed drug resistance. According to the WHO-approved classification of drug-resistant TB [31, 32], both isolates of six patients and the second *Mtb* sample of P42SIT1.17 were isoniazid-resistant (HR). Both isolates acquired from nine individuals and initial *Mtb* samples of P42SIT1.17 and P46SIT1.19 patients, were MDR, while both isolates of five patients were pre-XDR. In one case, transition from HR to MDR, and in four more cases, transition from MDR to pre-XDR occurred between subsequently acquired *Mtb* isolates.

All studied SIT40, SIT47, SIT156, and SIT254 isolates, and most (12/18, 66.7%) SIT53 *Mtb* isolates were drug susceptible. The largest diversity in drug resistance type was observed for SIT1 isolates. MDR and pre-XDR isolates belonged to the SIT1, SIT190, SIT42, and SIT1117 spoligotypes, while the HR type was detected in the SIT1, SIT283, and SIT53 isolates. The association of isolate drug resistance with its spoligotype was statistically significant (χ^2^_(4; 104)_ = 24.07, df=4, *P*<0.001). Drug susceptibility was distributed among SIT254 isolates with greater observed frequency than expected (standardized residuals >4), while drug resistance was more common for SIT1 (standardized residuals>3). SIT42, SIT53 and rare spoligotype pathogen drug susceptibility or resistance were equally expected. The Cohen’s W value indicated a moderately strong association between these categories (0.48, 95 % CI 0.37–0.63). At the same time, observations provided no more than 501.56 times greater support for the existing association between drug resistance and *Mtb* sample spoligotype (*H_A_*) than its absence (*H_0_*).

Drug-susceptible and HR isolates were more common in the recurrent TB patient group, while MDR and pre-XDR isolates prevailed among longitudinal single TB episode isolates. A statistically significant association was found between pathogen drug resistance and patient cohort (χ^2^_(1; 104)_ = 4.87, df=1, *P*=0.03). Drug-resistant longitudinal *Mtb* isolates of a single TB episode were more frequent than expected. At the same time, drug-susceptible samples were more common in the recurrent TB-experienced patient group (standardized residuals >2). However, according to the Cohen's W value, this association was weak (0.24, 95 % CI 0.05–0.42), and observations provided no more than 3.74 times greater support for *H_A_*.

In addition, no statistically significant association was found between patient cohort and sample spoligotype (χ^2^_(4; 104)_ = 8.84, df=4, *P*=0.07). The Cohen’s W value indicated a weak association between variables (0.29, 95 % CI 0.18–0.49), and observations provided no more than only 2.07 times bigger support for *H_A_*.

### Longitudinal single-episode *Mtb* isolates

**Fig. 2.**
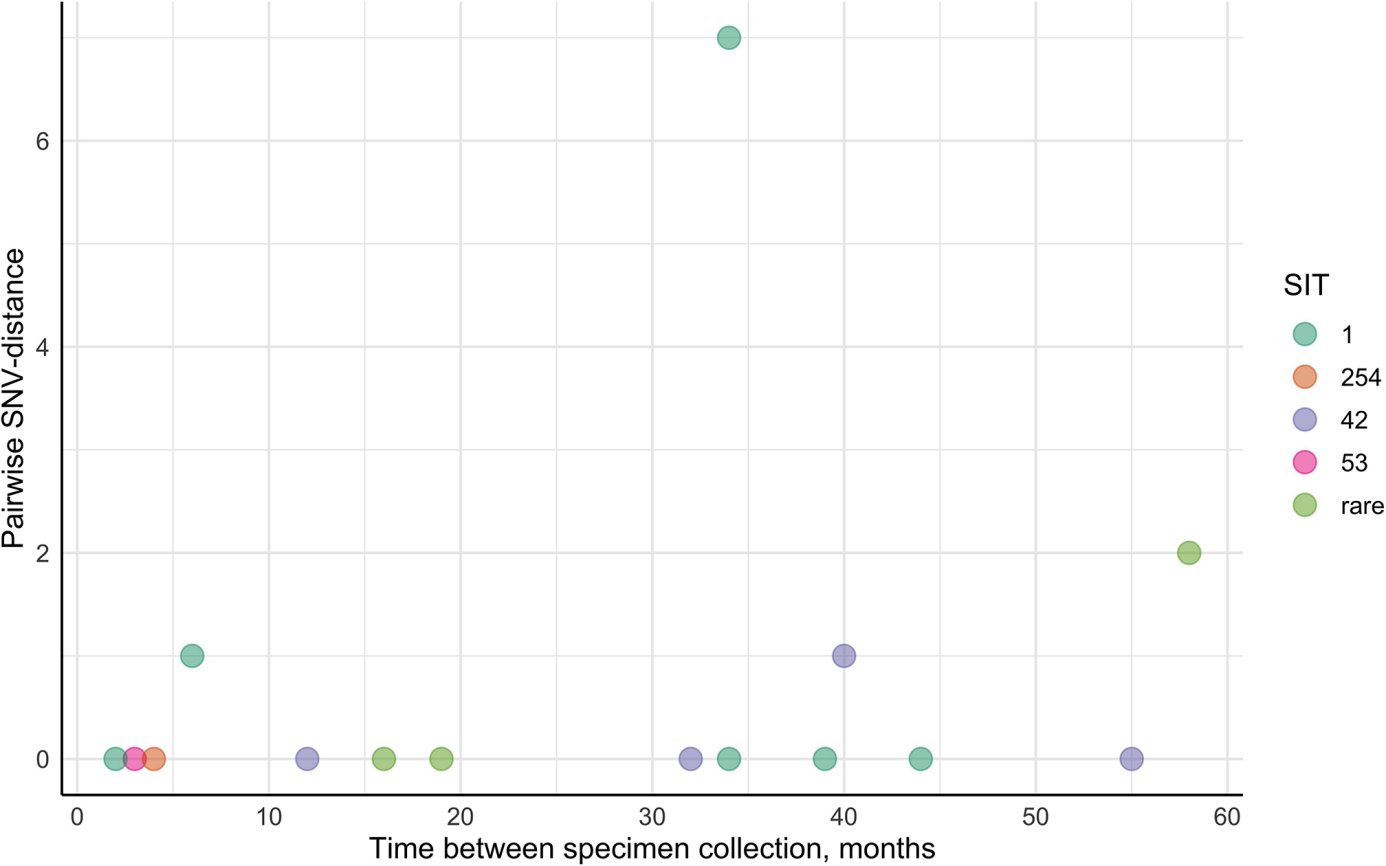
Pairwise SNV-distance differences against the time interval between specimen collection in the longitudinal single TB episode isolate group.

Isolates acquired from each patient were analysed in pairs to calculate pairwise SNV distances and identify differing SNVs for each sample. In total, 16 *Mtb* isolate pairs were obtained from patients who experienced a single TB episode of different durations (Fig. 2, Table 2). In this group, the time between specimen collection varied from 2 to 58 months. Eleven sample pairs had the same SNV pattern regardless of the time between episodes; in three cases, mycobacteria acquired 1 SNV, while one patient’s second isolate gained 7 additional SNVs. For one patient, a differing SNV was found in the first isolate.

**Table 2.** Characteristics of the longitudinal single TB episode isolate group

Patient ID	SIT	first isolate		second isolate		Time between specimen collection, months	Pairwise SNV-distance
		no. of differing SNVs	Drug resistance type	no. of differing SNVs	Drug resistance type		
P1SIT1.1	1	0	pre-XDR	0	pre-XDR	34	0
P2SIT1.2	1	0	MDR	0	MDR	39	0
P24SIT1.9	1	0	S	0	S	44	0
P27SIT1.12	1	0	pre-XDR	7	pre-XDR	34	7
P28SIT42.5	42	0	MDR	0	MDR	55	0
P29SIT42.6	42	0	MDR	0	MDR	12	0
P34SIT190.1	190	0	pre-XDR	2	pre-XDR	58	2
P35SIT42.7	42	0	pre-XDR	0	pre-XDR	32	0
P36SIT1.13	1	1	S	0	S	12	1
P38SIT40.1	40	0	S	0	S	16	0
P43SIT283.1	283	0	HR	0	HR	19	0
P48SIT42.9	42	0	MDR	1	MDR	40	1
P49SIT53.9	53	0	S	0	S	3	0
P50SIT254.7	254	0	S	0	S	4	0
P51SIT1.20	1	0	MDR	0	MDR	2	0
P52SIT1.21	1	0	MDR	1	pre-XDR	6	1

HR, isoniazid-resistant; MDR, multidrug-resistant; pre-XDR, preextensively drug-resistant; S, drug-susceptible.

### Recurrent TB cases and determination of possible causes

**Fig. 3.**
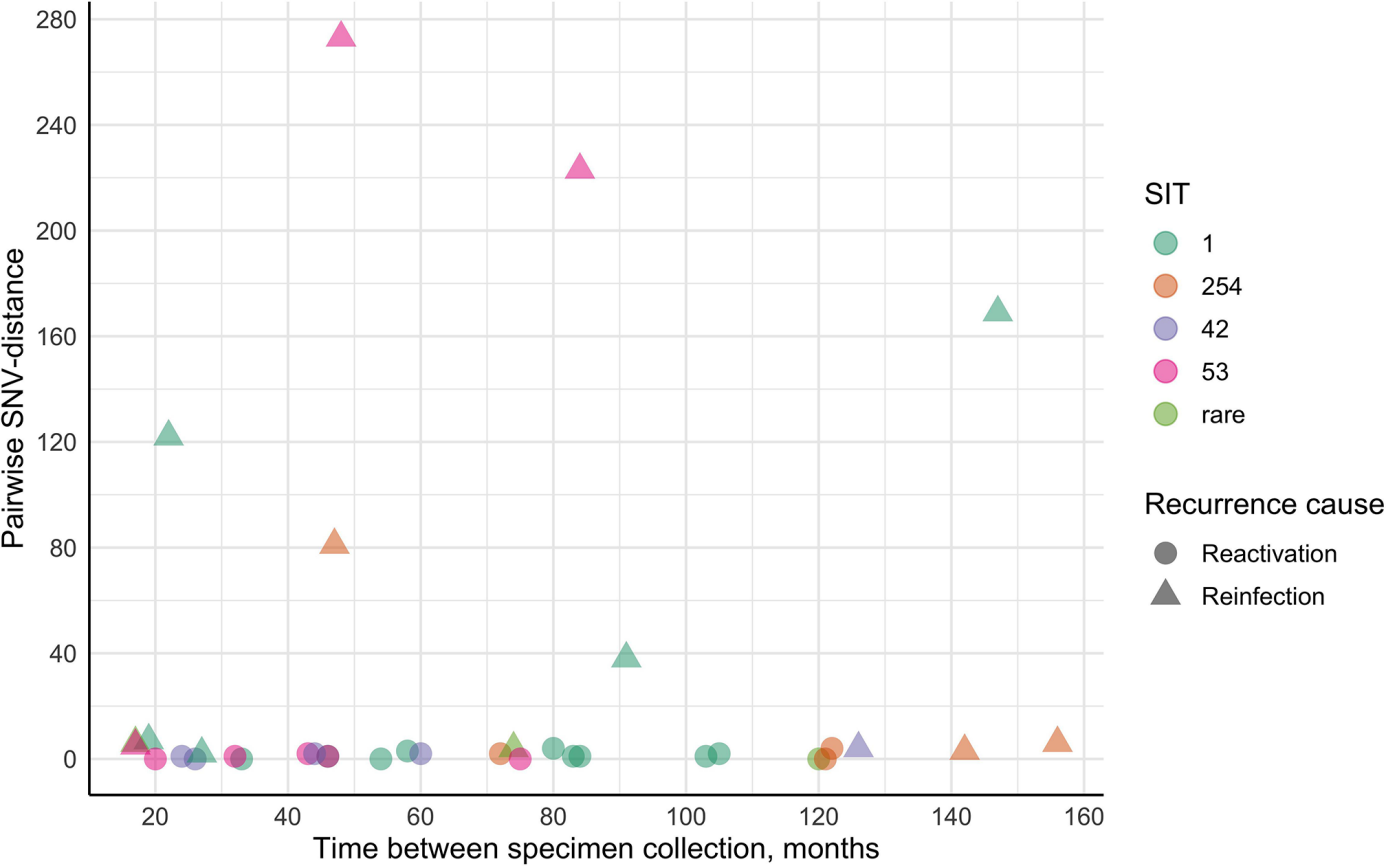
Pairwise SNV-distance differences against the time interval between specimen collection in recurrent TB patient isolates.

In total, 36 isolate pairs were included in the recurrent TB group (Fig. 3, Table 3). The time between specimen collection varied from 17 to 156 months. According to the pDST results, most patients in this cohort either had identical drug resistance profiles or their later isolates were resistant to more antimicrobials and therefore had more severe drug resistance. Second isolates of six recurrent TB patients (P25SIT1.10, P26SIT1.11, P37SIT1.14, P46SIT1.19, P47SIT42.8) developed additional resistance(s), as pDST data suggested. Only one studied case of recurrent TB (P42SIT1.17) could be classified as reinfection before sequencing data were available because the first isolate showed more severe drug resistance than the second isolate (MDR vs HR).

**Table 3.** Characteristics of the recurrent TB isolate group

Patient ID	SIT	First isolate		Second isolate		Time between specimen collection, months	Pairwise SNV-distance	Identified cause of recurrence
		no. of differing SNVs	Drug resistance type	no. of differing SNVs	Drug resistance type			
P3SIT254.1	254	0	S	2	S	72	2	Reactivation
P4SIT1.3	1	0	S	0	S	33	0	Reactivation
P5SIT1117.1	1117	0	MDR	0	MDR	120	0	Reactivation
P6SIT53.1	53	0	S	2	S	43	2	Reactivation
P7SIT53.2	53	102	S	121	S	84	223	Reinfection
P8SIT1.4	1	0	S	3	S	58	3	Reactivation
P9SIT42.1	42	1	S	3	S	126	4	Reinfection
P10SIT42.2	42	0	S	0	S	26	0	Reactivation
P11SIT1.5	1	2	S	5	S	19	7	Reinfection
P12SIT53.3	53	141	S	132	S	48	273	Reinfection
P13SIT254.2	254	40	S	41	S	47	81	Reinfection
P14SIT254.3	254	0	S	0	S	121	0	Reactivation
P15SIT254.4	254	0	S	4	S	122	4	Reactivation
P16SIT1.6	1	0	MDR	0	MDR	54	0	Reactivation
P17SIT156.1	156	5	S	1	S	17	6	Reinfection
P18SIT47.1	47	2	S	2	S	74	4	Reinfection
P19SIT42.3	42	0	S	2	S	60	2	Reactivation
P20SIT53.4	53	0	S	0	S	20	0	Reactivation
P21SIT1.7	1	0	S	1	S	84	1	Reactivation
P22SIT42.4	42	0	MDR	1	MDR	24	1	Reactivation
P23SIT1.8	1	1	HR	1	HR	27	2	Reinfection
P25SIT1.10	1	0	MDR	1	pre-XDR	46	1	Reactivation
P26SIT1.11	1	0	MDR	1	pre-XDR	83	1	Reactivation
P30SIT53.5	53	0	S	0	S	75	0	Reactivation
P31SIT53.6	53	0	HR	1	HR	46	1	Reactivation
P32SIT53.7	53	0	HR	1	HR	32	1	Reactivation
P33SIT53.8	53	1	HR	4	HR	17	5	Reinfection
P37SIT1.14	1	18	HR	20	MDR	91	38	Reinfection
P39SIT1.15	1	0	HR	1	HR	103	1	Reactivation
P40SIT254.5	254	2	S	4	S	156	6	Reinfection
P41SIT1.16	1	0	pre-XDR	2	pre-XDR	105	2	Reactivation
P42SIT1.17	1	88	MDR	81	HR	147	169	Reinfection
P44SIT254.6	254	1	S	2	S	142	3	Reinfection
P45SIT1.18	1	0	MDR	4	MDR	80	4	Reactivation
P46SIT1.19	1	61	S	61	MDR	22	122	Reinfection
P47SIT42.8	42	0	MDR	2	pre-XDR	44	2	Reactivation

HR, isoniazid-resistant; MDR, multidrug-resistant; pre-XDR, preextensively drug-resistant; S, drug-susceptible.

Based on the WGS results, the pairwise SNV-distance in six isolate pairs (6/36, 16.7%) was between 38 and 273 SNVs; therefore, reinfection was the apparent cause of TB recurrence for these individuals, including patient P42SIT1.17 (pairwise SNV-distance = 169) and patients P37SIT1.14 and P46SIT1.19, whose later isolate was more resistant than the initial isolate. However, in most cases (30/36, 83.3%), SNV-distance was less than 10 SNVs for all spoligotypes regardless of the interval duration. In seven pairs, the *Mtb* first and second samples were identical, and in 15 more cases, the second isolate gained 1-4 additional SNVs, which indicates possible endogenous reactivation (22/36, 61.1%). Isolates of eight patients (22.2%) had a relatively small pairwise SNV-distance of 2-7; however, in all these patients, the initial isolate had 1–5 SNVs that were not found in subsequent *Mtb* samples either in any coverage or in at least 4-read coverage. Otherwise, the finding was not considered as differing SNV. For these patients, TB recurrence likely occurred due to reinfection.

**Fig. 4.**
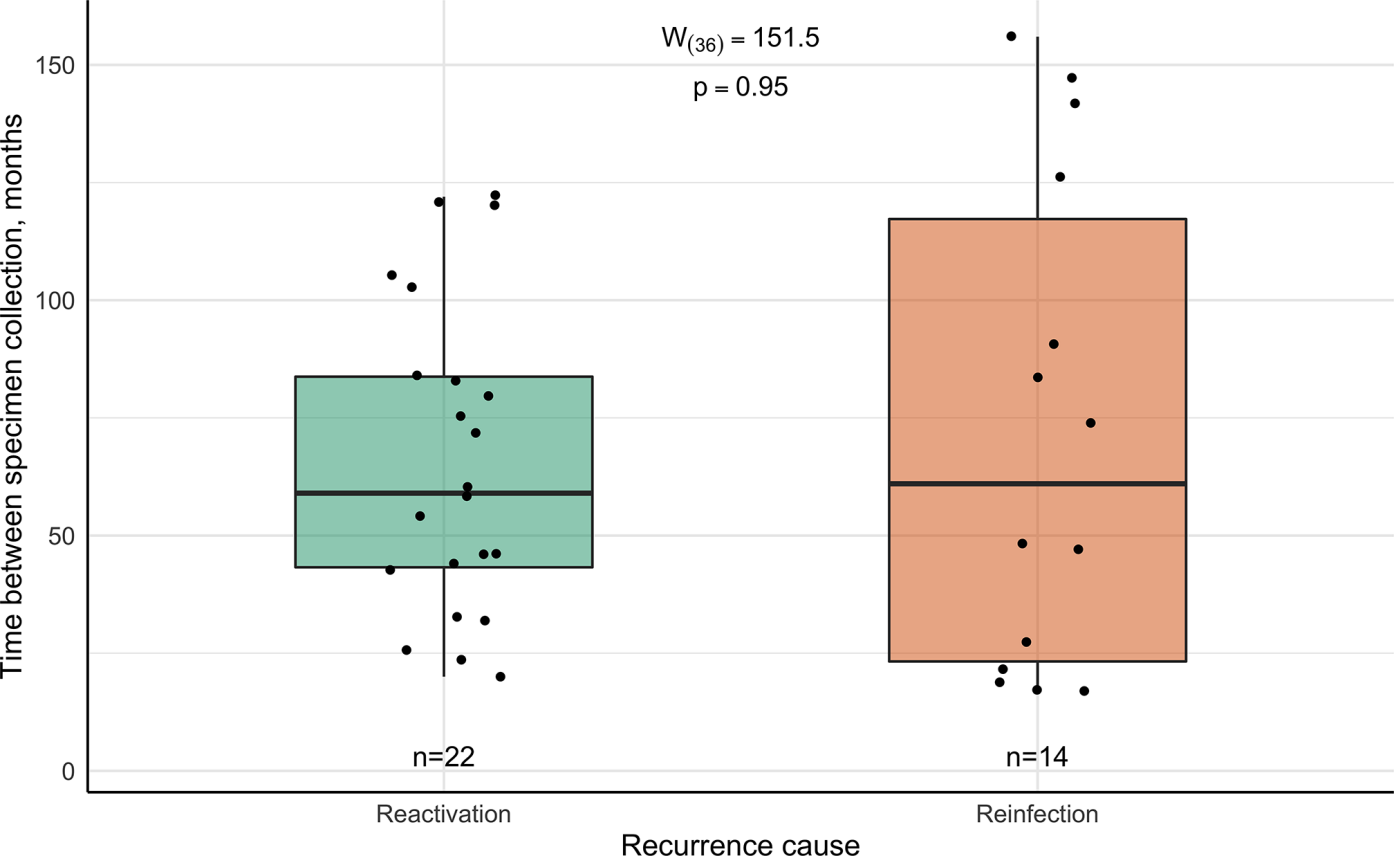
Dispersion of the time interval between episodes in possible reactivation and reinfection cohorts.

Differences in the time interval between specimen collection in patient cohorts divided by the most likely cause of TB recurrence (possible reactivation and reinfection) were analysed. Slight data asymmetry and no outliers were observed, and a normal distribution could not be rejected in either the possible reactivation (W_(22)_ = 0.93, *P*=0.13) or reinfection groups (W_(14)_ = 0.87, *P*=0.05). The median time between specimen collection was 59 months (IQR 43.3–83.8) for possible TB reactivation-experienced patients and 61 months (IQR 23.3–117.3) in the reinfection group (Fig. 4). No statistically significant difference was found between these groups (W_(36)_ = 151.5, *P*=0.95). A minor, negligible positive effect (0.01, 95 % CI 0.006–0.41) was observed, suggesting that the time interval between episodes tends to be slightly longer in the reactivation group. Observations provided no more than only 7.29 times greater support for the existing differences between studied cohorts (*H_A_*) than the presumption that there are none (*H_0_*). Based on the results of the performed test and considering the wide dispersion of this variable in both cohorts it could be presumed that it is equally possible for an individual to obtain either of the recurrent TB forms in various periods since the first TB episode was cured.

In addition, no statistically significant association between deciphered recurrent TB episode cause and *Mtb* drug resistance was determined (χ^2^_(1; 36)_ = 0.25, df=1, *P*=0.62). Observations provided no more than 1.24 times more support for *H_A_*, meaning that association and its absence between these variables are equally possible, while a small statistically nonsignificant Cohen's W value (0.14, 95 % CI 0–0.47) accepted that association is weak. Fisher’s exact test did not demonstrate any statistically significant association between recurrence cause and isolate spoligotype (*P*=0.79).

### Analysis of recently acquired SNVs

Isolates from both patient cohorts, i.e., longitudinal single-episode TB cases and recurrent TB cases, were included, and 77 SNVs from 36 *Mtb* isolates were analysed (Supplementary material 2). In total, 88.3 % (68/77) of the SNVs were detected in protein-coding genes, and 11.7 % (9/77) were found in intergenic regions.

No pattern of variant accumulation in particular genes could be identified, as in most cases, SNVs accumulated randomly in various positions throughout the whole mycobacterial genome. Two drug-resistant samples (P39SIT1.15-03.10.2019 and P48SIT42.9-23.05.2019) had differing SNVs in the locus Rv0131c (*fadE1* gene), while four more subsequent isolates (P25SIT1.10-08.02.2008, P26SIT1.11-03.03.2011, P52SIT1.21-15.04.2010, and P47SIT42.8-26.10.2011) gained additional SNVs in Rv0006 (*gyrA* gene), which induced fluoroquinolone (FQ) resistance development. More specifically, an Asp94Gly mutation was detected in three SIT1 isolates, while Gly88Cys was found in the SIT42 isolate. This finding was confirmed by pDST data, which also showed the transition from MDR to pre-XDR type for these isolate pairs.

Missense and synonymous variants in protein-coding genes were present with similar frequencies [48.5 % (33/68) and 47.1 % (32/68), respectively], while stop mutations were found only in 4.4 % (3/68) of cases. The occurrence distribution of SNV effects was significantly different from the predicted distribution (χ^2^_(2; 68)_ = 25.62, df=2, *P*<0.001). Standardized residuals indicated that stop mutations (standardized residual <-5) were significantly less common than expected, while both synonymous and missense variants occured more frequently (standardized residuals>2). The observations provide no more than 10 493.67 times greater support for the uneven distribution of accumulated SNV effects (*H_1_*) than for assuming that all effects occur with equal frequency (*H_0_*). The Cohen’s W value is 0.61, which implies a large effect magnitude and therefore indicates a partial matching of the results with the SNV functional effect distribution in the population.

### Mycobacterial mutation rate

**Fig. 5.**
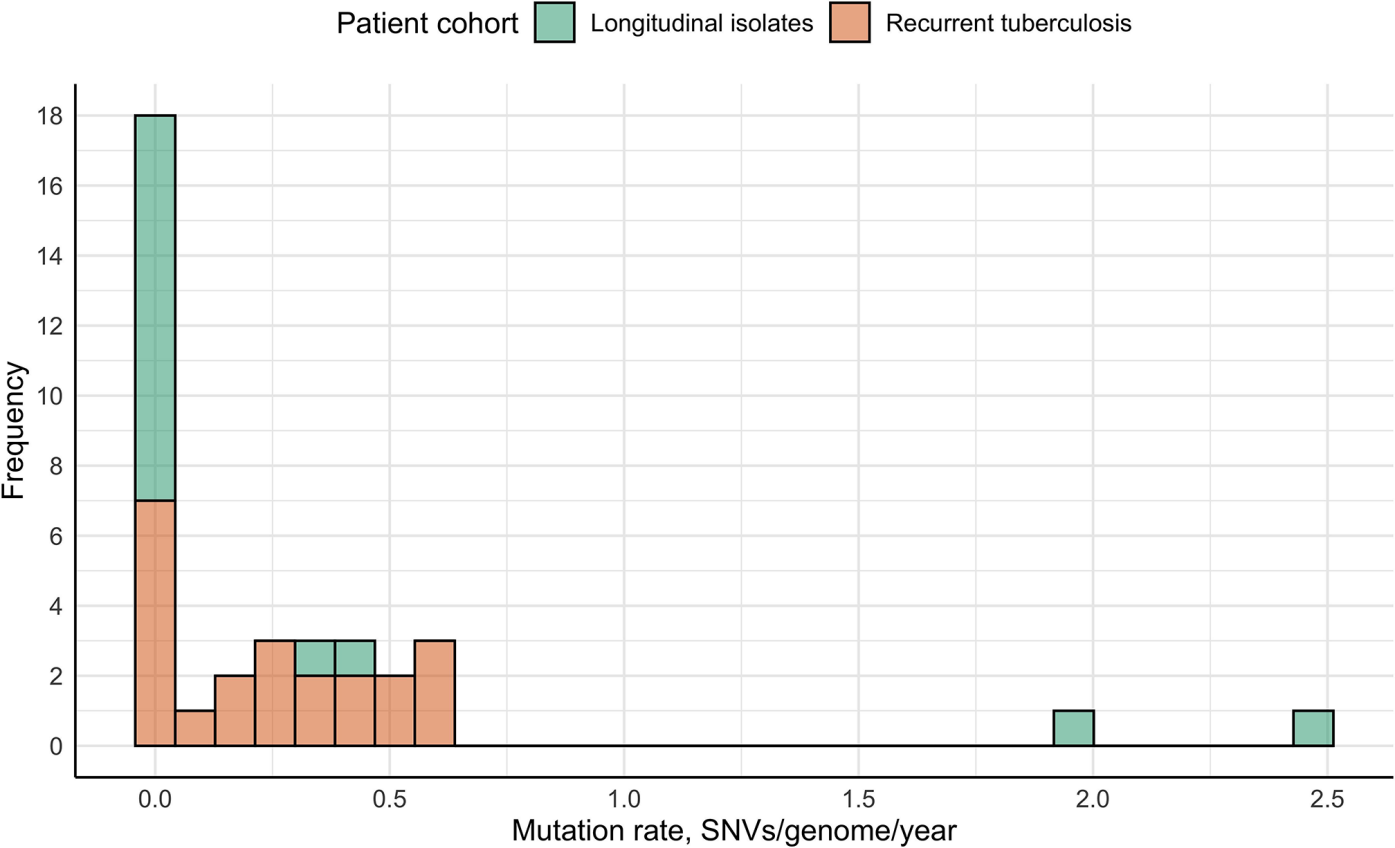
Calculated mycobacterial mutation rate in longitudinal single TB episodes and recurrent TB (possible reactivation) isolates.

The mycobacterial mutation rate was calculated as SNVs per genome per year for longitudinal single TB episodes, and possible reactivation isolate pairs (*n*=37; Fig. 5), including four cases of acquired FQ resistance. Overall, the median mutation rate was 0.12 SNVs/genome/year (IQR 0–0.39). In 18 cases (48.6 %), zero SNVs accumulated; additionally, two outlier values of 2 and 2.47 SNVs/genome/year were observed (5.4%), while in the remaining 17 isolate pairs (45.9%), the mutation rate varied from 0.12 to 0.62 SNVs/genome/year with a mean value of 0.36±0.16 SNVs/genome/year.

**Fig. 6.**
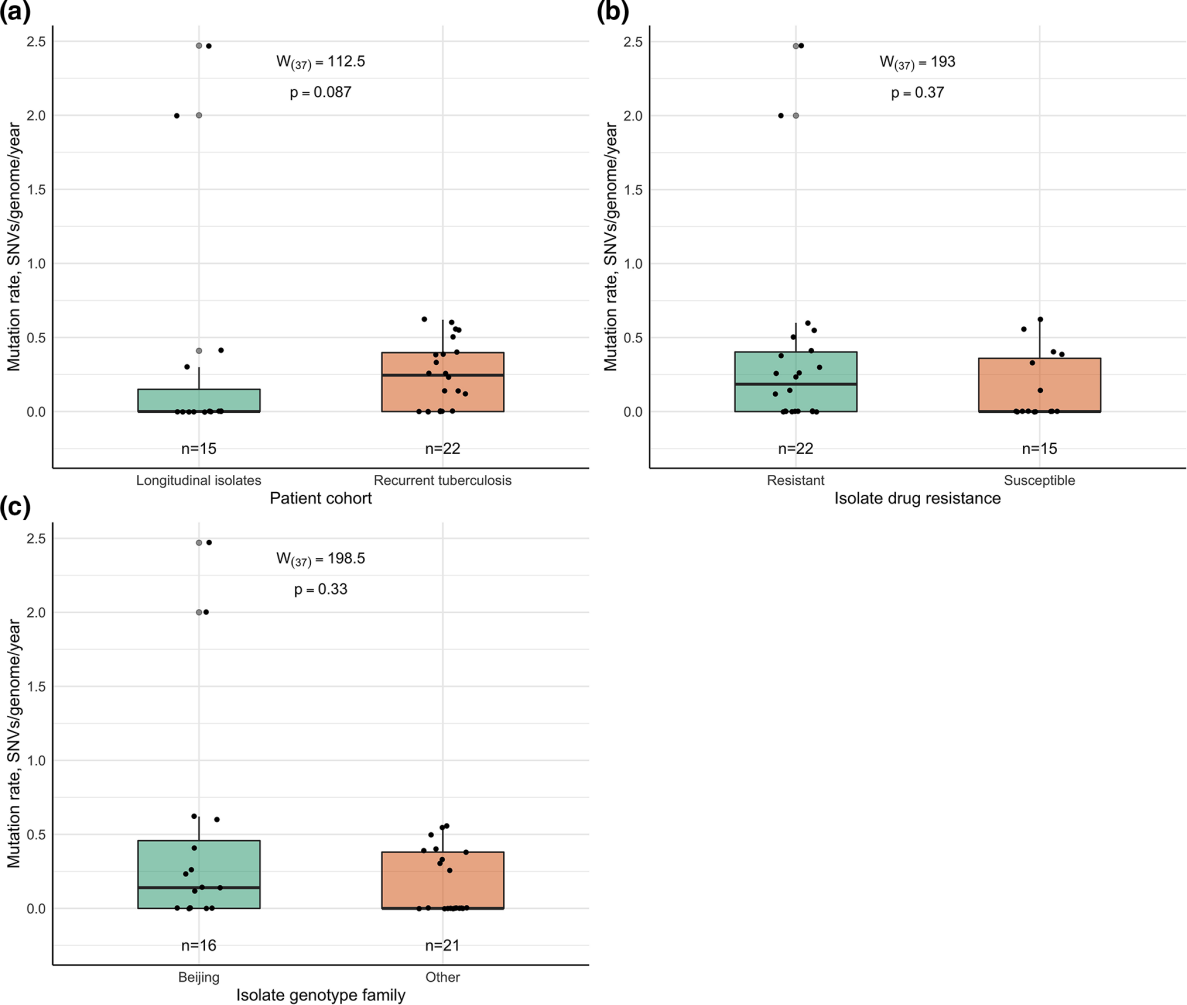
Differences in mycobacterial mutation rate distribution in both patient cohorts (**a**), drug-susceptible and resistant isolates (**b**), as well as in isolates of the Beijing and other genotype families (**c**).

Differences in *Mtb* mutation rate were also assessed in isolate groups defined by patient cohort, drug resistance status, and *Mtb* genotype family (Table 4, Fig. 6). Two outliers were drug-resistant SIT1 isolate pairs of the longitudinal single TB episode cases. Overall, the highest prevalence of subsequent samples that accumulated zero additional SNVs was found in the longitudinal single-episode isolate group (11/15, 73.3 %). At the same time, the highest mutation rate of 0.62 SNVs/genome/year, excluding outliers, was observed in drug-susceptible recurrent TB cases caused by mycobacteria of the Beijing family (SIT1).

**Table 4.** Mycobacterial mutation rate values in different isolate groups

Variable		Patient cohort		Drug resistance status		Genotype family	
		Longitudinal isolates (*n*=15)	Recurrent TB (*n*=22)	Resistant (*n*=22)	Susceptible (*n*=15)	Beijing (*n*=16)	Other (*n*=21)
Mutation rate (SNVs/ genome /year)	Median	0	0.25	0.19	0	0.14	0
	IQR	0–0.15	0–0.4	0–0.4	0–0.36	0–0.46	0–0.38
	Maximum	0.41*	0.62	0.6*	0.62	0.62*	0.56
Number of samples acquired 0 SNVs		11 (73.3 %)	7 (31.8 %)	9 (40.9 %)	9 (60 %)	6 (37.5 %)	12 (57.1 %)

*Outliers with mutation rates of 2 and 2.47 SNVs/genome/year were excluded.

In all sample groups excluding resistant *Mtb* isolates, an asymmetric distribution of mutation rate was observed. Outlier values were present in the longitudinal single TB episode isolate group, and among resistant and Beijing isolates (SIT1 and SIT190). Normal distribution could be rejected in both patient cohorts (W_(15)_ = 0.51, *P*<0.001; W_(22)_ = 0.88, *P*=0.014), isolate groups divided by resistance status (W_(22)_ = 0.61, *P*<0.001; W_(15)_ = 0.73, *P*<0.001) and genotype family (W_(16)_ = 0.64, *P*<0.001; W_(21)_ = 0.75, *P*<0.001). No statistically significant differences in the mycobacterial mutation rate were found between longitudinal single TB episodes and possible reactivation groups (W_(37)_ = 112.5, *P*=0.087), drug-resistant and susceptible *Mtb* samples (W_(37)_ = 193, *P*=0.37) or isolates of Beijing and other genotype families (W_(37)_ = 198.5, *P*=0.33). In all cases, the Bayes factor upper bound was approximately 1, meaning that *H_0_* and *H_A_* are equally possible. A small positive effect showed that the mutation rate tended to be slightly faster in the longitudinal single-episode (0.28, 95 % CI 0.02–0.61), drug-resistant (0.15, 95 % CI 0.01–0.46) and Beijing genotype isolate groups (0.16, 95 % CI 0.01–0.47).

## Discussion

In this study, we analysed all available *Mtb* isolate pairs of the same spoligotype acquired in 17-year period from Latvian TB patients who either had issues reaching successful treatment outcomes during a single episode or had experienced a recurrence of the disease. Most isolate pairs had the SIT1 spoligotype, followed by SIT42, SIT254 and SIT53, which are highly prevalent in Latvia [24], and greater genetic relatedness of isolates within the SIT1, SIT42, and SIT254 spoligotypes were demonstrated in the created phylogeny. Along with different *Mtb* genotypes, a wide variety of drug resistance types were present among the studied isolates as well, and more than half of the individuals were infected with drug-resistant strains. Our data confirmed the common opinion that drug resistance is highly distributed among the Beijing genotype (SIT1) strains [33–35], particularly in the investigated geographical region [36]. However, in contradiction to previous studies that reported an association between the *Mtb* LAM family and drug resistance [37–39], this study showed that SIT254 (LAM-RUS) strains were more prone to be therapy-susceptible. This finding highlights possible differences in predisposition to drug resistance development between *Mtb* clades and within genotype families. Acquired data also suggest that some spoligotypes (i.e., SIT1, SIT42) were more likely to develop MDR or pre-XDR, while others were usually drug-susceptible or had milder resistance (such as SIT53, SIT254); however, further research is needed to confirm this observation.

Although spoligotypes were distributed similarly in both patient cohorts, we found that isolate drug susceptibility was more common in the recurrent TB patient group, while *Mtb* drug resistance was more expected for those patients who experienced a single, in most cases – prolonged TB episode. Notably, the latter cohort comprised patients with a history of noncompliance with therapy, withdrawal and abacilation difficulties. Although the treatment strategy for highly drug-resistant TB has improved over recent years, there is still a risk of poor outcomes and arbitrary discontinuation due to severe adverse effects of the medications used [40–43]. Moreover, the Beijing genotype was prevalent among the drug-resistant isolates in this study and is still frequently associated with treatment failure cases [44].

The most important part of this study was determining recurrent TB causes and distinguishing between reinfection and endogenous reactivation cases. The previously proposed algorithm [13, 14] uses SNV distances between the initial and subsequent isolates, implying that separation of more than 100 SNVs indicates reinfection. This study used two consecutive approaches to decipher the cause of recurrent TB episodes: the detection of pairwise SNV distances and the identification of differing SNVs between paired isolates. Only 6 of 36 cases (16.6%) could be classified as reinfection based on a pairwise SNV-distance, and in two cases, the separation between two isolates was less than 100 SNVs (38 and 81 SNVs). As only those TB recurrences that were caused by the same *Mtb* spoligotype as the initial episode were studied, we presume that in the case of reinfection with a closely related strain, SNV-distance can be highly variable and dependent on the prevalence of the particular genotype in the studied geographical territory. Interestingly, the genetic distance between paired SIT53 isolates was noticeably higher (223 and 273 SNVs) than in the SIT1 (38-169 SNVs) and SIT254 (81 SNVs) isolates. Moreover, the phylogenetic analysis demonstrated greater genetic diversity between the studied SIT53 isolates than other common genotypes in the studied area (i.e., SIT1, SIT42, and SIT254).

For the most recurrent TB isolate pairs (30/36, 83.3%), SNV-distance did not exceed 10 SNVs; therefore, these cases could be classified as endogenous reactivation based on proposed SNV-distance thresholds of 6-10 SNVs from various studies [12–14]. When considering differing SNVs for both isolates in each pair, we found that in eight cases, the first isolate had a low number of SNVs (range 1–5 SNVs) that were not present in the later *Mtb* sample. Similarly, the same issue was observed for one patient in the longitudinal single TB episode cohort (P36SIT1.13). In all cases, except for P36SIT1.13, the latter isolate also harboured 1–5 differing SNVs. There is a possibility that novel mutations accumulated during the *Mtb* cultivation process in the laboratory; however, the results of Brown *et al*. and Genestet *et al*. studies [45, 46] demonstrated that there were no significant variations between direct sequencing data of sputum specimens and WGS results acquired from the cultured *Mtb* isolates. Furthermore, a mutation loss is also unlikely because SNVs tend to accumulate in the direction of disease transmission [47]. Based on this data, we suggest that these eight patients were either involved in a local TB outbreak or were in close contact with other individuals harbouring an active TB infection caused by the same *Mtb* strain and thus were reinfected. At the same time, the possibility that paired specimens could represent different portions of patient’s lungs, therefore explaining small genetic distance between the acquired isolates, could not be ignored, and this hypothesis needs to be studied further.

According to the sequencing data, most recurrent TB patients (22/36, 61.1%) experienced another episode due to endogenous reactivation, as either isolates were identical, or all differing SNVs were detected in the subsequent isolate. However, the results of recently conducted studies showed [19–21] that recurrent TB cause determination is more precise if detection of genetic distances is being complemented with delineation of the transmission network. This approach is based on clustering *Mtb* isolates acquired from all known TB patients potentially involved in the same transmission chain. It allows the identification of reinfection cases with clonal or closely related strains. The limitation of our study is the lack of contact tracing information and sequencing data. Therefore, we classify previously described cases as “possible reactivation”. Previous data suggested that endogenous reactivation is often associated with *Mtb* drug resistance [13], and reinfection tends to occur within a longer time frame than reactivation [20]. No differences in drug resistance status, spoligotype pattern, or time between episodes were found for identified reactivation, and reinfection isolate pairs; therefore, we presume that these criteria could not always help decipher the cause of recurrent TB episodes.

Mycobacterial mutation rate is another *Mtb* strain infection describing parameter since WGS has become the most common approach in TB studies. It demonstrated great variability among longitudinal single TB episodes and possible reactivation isolates. If no outlier and zero values were considered, the average mutation rate was 0.36 SNVs/genome/year, which corresponded to the previously determined value of 0.3–0.5 SNVs/genome/year in several TB transmission studies [17, 48, 49]. However, since the observed amplitude of 0.12–0.62 SNVs/genome/year was wide, an impact of various exogenous factors is suspected. Unfortunately, due to the limited sample set, we could not find any statistically significant differences in mutation rate between the studied patient cohorts, drug-resistant and susceptible *Mtb* samples or isolates of the Beijing and other families; therefore, no possible mutagenesis-lowering or mutagenesis-enhancing factors could be identified. Simultaneously, two outlier values of 2 and 2.47 SNVs/genome/year were observed for two drug-resistant SIT1 isolate pairs within the single TB episode cohort. Although recently conducted studies showed that mutagenesis of drug-resistant isolates [48], as well as *Mtb* of Beijing family tends to be much faster, reaching up to 18 new SNVs per year [50], based on our data, we suppose that the high mutation rate of the mentioned isolates was also associated with different host- and therapy-related factors as patient characteristics, therapy compliance, and treatment regimen, as suggested previously [11]. Moreover, it remains unclear how the *Mtb* mutation rate changes if bacteria are dormant, as available data are controversial: some results show that *Mtb* mutates at a similar rate during latency [51, 52], while another study demonstrated that mutagenesis during latent infection is approximately 10 times slower than in the active state [53]. In our study, nearly half of the subsequent isolates did not accumulate any new SNVs or acquired a lower number of SNVs than expected based on the proposed average mutation rate regardless of time, which may indicate a latency period. In cases of recurrent TB, this statement provides additional support for the possibility of endogenous reactivation rather than reinfection. However, we believe that mutation rate should not be used alone to predict the cause of TB recurrence due to the lack of information on its variability.

Furthermore, no significant SNV accumulation pattern could be detected in the studied *Mtb* isolates regardless of the patient cohort. However, sequencing data allowed us to detect FQ resistance development through one accumulated SNV in a subsequent isolate of three recurrent TB patients and one single TB episode patient. FQ are essential components of drug-resistant TB treatment regimens [31], and understanding the reason for selective FQ resistance acquisition is essential. Some drug resistance development stimulating factors, such as noncompliance with therapy and withdrawal, are known [54], however, unlike most anti-TB agents, FQ is widely used for treating other respiratory, urogenital, and gastrointestinal infections [55]. Rapid *Mtb* FQ resistance development 13 days after the onset of empirical treatment of yet unknown aetiology prostatitis was reported previously [56]. In that case, prescribed therapy (levofloxacin, ethambutol, and clarithromycin) was insufficient to eradicate later diagnosed *Mtb* infection, thought to be a cause of resistance development. It also suggested that resistance to FQ develops more easily under antibiotic selection pressure conditions than other anti-TB drugs, as subsequent isolates of that patient did not acquire ethambutol resistance. Moreover, empirical use of FQ drugs for pneumonia treatment was also associated with a longer delay in pulmonary TB diagnosis and treatment [57]. In our study, the reason for FQ resistance acquisition remains unclear for recurrent TB patients, as all of them completed the first TB episode therapy, and their medical records between the initial FQ-susceptible TB episode and the relapse of FQ-resistant *Mtb* infection were not specified. However, the previously described phenomenon of rapid FQ resistance acquisition due to incomplete treatment could explain our single TB episode patient case, who arbitrarily discontinued therapy and whose second FQ-resistant isolate was obtained 6 months after initial diagnosis.

In summary, this study highlighted high variability in SNV distance and precise cause determination complexity for recurrent TB episodes caused by the same *Mtb* genotype as initial active TB infection. It showed the usefulness of differing SNV detection for *Mtb* isolates in such cases. Drug-susceptible isolates were more common in the recurrent TB patient group, while no differences in time between episodes or distribution of drug resistance or spoligotype patterns were found between the identified possible reactivation and reinfection cases.

## Supplementary Data

Supplementary material 1
