# Supplementary material for: Advantages of analysing both pairwise SNV-distance and differing SNVs between Mycobacterium tuberculosis isolates for recurrent tuberculosis cause determination
Source: Microb Genom. 2023 Mar 23;9(3):mgen000956. doi: 10.1099/mgen.0.000956 (PMC10132068; doi:10.1099/mgen.0.000956)
Supplement: Supplementary material 1 [file mgen-9-956-s001.pdf]

Supplementary material 1. Assigned patient and isolate identification codes.

Patient ID	Sample ID	Phylogeny ID	Sequencing ID
P1SIT1.1	P1SIT1.1-16.10.2012	SIT1.1-16.10.2012	LVE2294
	P1SIT1.1-04.08.2015	SIT1.1-04.08.2015	LVE4941
P2SIT1.2	P2SIT1.2-27.09.2002	SIT1.2-27.09.2002	LVE2446
	P2SIT1.2-04.01.2006	SIT1.2-04.01.2006	LVE6674
P3SIT254.1	P3SIT254.1-03.04.2008	SIT254.1-03.04.2008	LVE9222
	P3SIT254.1-14.04.2014	SIT254.1-14.04.2014	LVE0321
P4SIT1.3	P4SIT1.3-10.05.2011	SIT1.3-10.05.2011	LVE3649
	P4SIT1.3-24.02.2014	SIT1.3-24.02.2014	LVE5176
P5SIT1117.1	P5SIT1117.1-16.02.2004	SIT1117.1-16.02.2004	LVE3316
	P5SIT1117.1-14.02.2014	SIT1117.1-14.02.2014	LVE4338
P6SIT53.1	P6SIT53.1-14.10.2010	SIT53.1-14.10.2010	LVE5578
	P6SIT53.1-23.05.2014	SIT53.1-23.05.2014	LVE3862
P7SIT53.2	P7SIT53.2-27.09.2007	SIT53.2-27.09.2007	LVE9645
	P7SIT53.2-06.10.2014	SIT53.2-06.10.2014	LVE5004
P8SIT1.4	P8SIT1.4-26.08.2009	SIT1.4-26.08.2009	LVE9644
	P8SIT1.4-02.07.2014	SIT1.4-02.07.2014	LVE7090
P9SIT42.1	P9SIT42.1-15.10.2003	SIT42.1-15.10.2003	LVE5167
	P9SIT42.1-15.04.2014	SIT42.1-15.04.2014	LVE0504
P10SIT42.2	P10SIT42.2-24.02.2010	SIT42.2-24.02.2010	LVE4833
	P10SIT42.2-12.04.2012	SIT42.2-12.04.2012	LVE2054
P11SIT1.5	P11SIT1.5-13.02.2013	SIT1.5-13.02.2013	LVE4960
	P11SIT1.5-24.09.2014	SIT1.5-24.09.2014	LVE3953
P12SIT53.3	P12SIT53.3-18.08.2010	SIT53.3-18.08.2010	LVE0670
	P12SIT53.3-27.08.2014	SIT53.3-27.08.2014	LVE1809
P13SIT254.2	P13SIT254.2-14.01.2010	SIT254.2-14.01.2010	LVE1158
	P13SIT254.2-01.12.2014	SIT254.2-01.12.2014	LVE0469
P14SIT254.3	P14SIT254.3-20.10.2004	SIT254.3-20.10.2004	LVE2150
	P14SIT254.3-21.11.2014	SIT254.3-21.11.2014	LVE9513
P15SIT254.4	P15SIT254.4-21.07.2004	SIT254.4-21.07.2004	LVE5134
	P15SIT254.4-24.09.2014	SIT254.4-24.09.2014	LVE3914
P16SIT1.6	P16SIT1.6-29.03.2010	SIT1.6-29.03.2010	LVE9441
	P16SIT1.6-24.09.2014	SIT1.6-24.09.2014	LVE3912
P17SIT156.1	P17SIT156.1-01.02.2013	SIT156.1-01.02.2013	LVE3603
	P17SIT156.1-01.07.2014	SIT156.1-01.07.2014	LVE6971
P18SIT47.1	P18SIT47.1-22.02.2008	SIT47.1-22.02.2008	LVE4691
	P18SIT47.1-17.04.2014	SIT47.1-17.04.2014	LVE0718
P19SIT42.3	P19SIT42.3-04.08.2009	SIT42.3-04.08.2009	LVE8058
	P19SIT42.3-24.07.2014	SIT42.3-24.07.2014	LVE9184
P20SIT53.4	P20SIT53.4-11.10.2012	SIT53.4-11.10.2012	LVE1934
	P20SIT53.4-17.06.2014	SIT53.4-17.06.2014	LVE6056
P21SIT1.7	P21SIT1.7-27.03.2007	SIT1.7-27.03.2007	LVE1991
	P21SIT1.7-10.03.2014	SIT1.7-10.03.2014	LVE6746
P22SIT42.4	P22SIT42.4-14.10.2011	SIT42.4-14.10.2011	LVE8357
	P22SIT42.4-25.10.2013	SIT42.4-25.10.2013	LVE0206
P23SIT1.8	P23SIT1.8-02.04.2009	SIT1.8-02.04.2009	LVE8788

	P23SIT1.8-21.06.2011	SIT1.8-21.06.2011	LVE2870
P24SIT1.9	P24SIT1.9-06.07.2006	SIT1.9-06.07.2006	LVE7363
	P24SIT1.9-18.03.2010	SIT1.9-18.03.2010	LVE6991
P25SIT1.10	P25SIT1.10-22.03.2004	SIT1.10-22.03.2004	LVE4317
	P25SIT1.10-08.02.2008	SIT1.10-08.02.2008	LVE9398
P26SIT1.11	P26SIT1.11-22.03.2004	SIT1.11-22.03.2004	LVE4318
	P26SIT1.11-03.03.2011	SIT1.11-03.03.2011	LVE6687
P27SIT1.12	P27SIT1.12-03.02.2005	SIT1.12-03.02.2005	LVE5514
	P27SIT1.12-27.11.2007	SIT1.12-27.11.2007	LVE8323
P28SIT42.5	P28SIT42.5-13.04.2012	SIT42.5-13.04.2012	LVE2313
	P28SIT42.5-08.11.2016	SIT42.5-08.11.2016	LVE5830
P29SIT42.6	P29SIT42.6-12.09.2007	SIT42.6-12.09.2007	LVE9025
	P29SIT42.6-08.09.2008	SIT42.6-08.09.2008	LVE9104
P30SIT53.5	P30SIT53.5-27.09.2005	SIT53.5-27.09.2005	LVE6303
	P30SIT53.5-22.12.2011	SIT53.5-22.12.2011	LVE5800
P31SIT53.6	P31SIT53.6-04.01.2010	SIT53.6-04.01.2010	LVE0020
	P31SIT53.6-04.11.2013	SIT53.6-04.11.2013	LVE4265
P32SIT53.7	P32SIT53.7-19.05.2005	SIT53.7-19.05.2005	LVE5847
	P32SIT53.7-15.01.2008	SIT53.7-15.01.2008	LVE9532
P33SIT53.8	P33SIT53.8-19.06.2009	SIT53.8-19.06.2009	LVE5346
	P33SIT53.8-24.11.2010	SIT53.8-24.11.2010	LVE2265
P34SIT190.1	P34SIT190.1-20.05.2010	SIT190.1-20.05.2010	LVE3000
	P34SIT190.1-05.03.2015	SIT190.1-05.03.2015	LVE4803
P35SIT42.7	P35SIT42.7-10.08.2005	SIT42.7-10.08.2005	LVE6115
	P35SIT42.7-22.04.2008	SIT42.7-22.04.2008	LVE9722
P36SIT1.13	P36SIT1.13-09.03.2011	SIT1.13-09.03.2011	LVE7199
	P36SIT1.13-20.03.2012	SIT1.13-20.03.2012	LVE3365
P37SIT1.14	P37SIT1.14-15.12.2011	SIT1.14-15.12.2011	LVE3053
	P37SIT1.14-26.07.2019	SIT1.14-26.07.2019	LVE7487
P38SIT40.1	P38SIT40.1-28.05.2018	SIT40.1-28.05.2018	LVE7046
	P38SIT40.1-12.09.2019	SIT40.1-12.09.2019	LVE0579
P39SIT1.15	P39SIT1.15-02.03.2011	SIT1.15-02.03.2011	LVE2705
	P39SIT1.15-03.10.2019	SIT1.15-03.10.2019	LVE2147
P40SIT254.5	P40SIT254.5-04.10.2006	SIT254.5-04.10.2006	LVE7511
	P40SIT254.5-27.09.2019	SIT254.5-27.09.2019	LVE1696
P41SIT1.16	P41SIT1.16-30.08.2010	SIT1.16-30.08.2010	LVE2441
	P41SIT1.16-24.05.2019	SIT1.16-24.05.2019	LVE2329
P42SIT1.17	P42SIT1.17-12.05.2003	SIT1.17-12.05.2003	LVE2273
	P42SIT1.17-21.08.2015	SIT1.17-21.08.2015	LVE2139
P43SIT283.1	P43SIT283.1-09.05.2013	SIT283.1-09.05.2013	LVE4070
	P43SIT283.1-18.12.2014	SIT283.1-18.12.2014	LVE4905
P44SIT254.6	P44SIT254.6-13.02.2008	SIT254.6-13.02.2008	LVE9434
	P44SIT254.6-28.11.2019	SIT254.6-28.11.2019	LVE6587
P45SIT1.18	P45SIT1.18-09.02.2005	SIT1.18-09.02.2005	LVE5436
	P45SIT1.18-28.09.2011	SIT1.18-28.09.2011	LVE6631
P46SIT1.19	P46SIT1.19-11.06.2009	SIT1.19-11.06.2009	LVE4727
	P46SIT1.19-12.04.2011	SIT1.19-12.04.2011	LVE2756
P47SIT42.8	P47SIT42.8-12.03.2008	SIT42.8-12.03.2008	LVE9583

	P47SIT42.8-26.10.2011	SIT42.8-26.10.2011	LVE2958
P48SIT42.9	P48SIT42.9-19.01.2016	SIT42.9-19.01.2016	LVE5486
	P48SIT42.9-23.05.2019	SIT42.9-23.05.2019	LVE2182
P49SIT53.9	P49SIT53.9-22.11.2010	SIT53.9-22.11.2010	LVE9358
	P49SIT53.9-23.02.2011	SIT53.9-23.02.2011	LVE2699
P50SIT254.7	P50SIT254.7-28.11.2008	SIT254.7-28.11.2008	LVE6400
	P50SIT254.7-25.03.2009	SIT254.7-25.03.2009	LVE1701
P51SIT1.20	P51SIT1.20-28.12.2009	SIT1.20-28.12.2009	LVE2092
	P51SIT1.20-02.03.2010	SIT1.20-02.03.2010	LVE3273
P52SIT1.21	P52SIT1.21-14.10.2009	SIT1.21-14.10.2009	LVE1301
	P52SIT1.21-15.04.2010	SIT1.21-15.04.2010	LVE1717

Supplementary material 2. Recently acquired SNVs in longitudinal single TB episode and recurrent TB isolate groups.

Sample ID	SIT	Genome position	Reference allele	Alternate allele	Locus tag	Gene	Product	AA change
P47SIT42.8-26.10.2011	42	7563	G	T	Rv0006	*gyrA*	DNA gyrase subunit A	Gly88Cys
P25SIT1.10-08.02.2008	1	7582	A	G	Rv0006	*gyrA*	DNA gyrase subunit A	Asp94Gly
P26SIT1.11-03.03.2011	1	7582	A	G	Rv0006	*gyrA*	DNA gyrase subunit A	Asp94Gly
P52SIT1.21-15.04.2010	1	7582	A	G	Rv0006	*gyrA*	DNA gyrase subunit A	Asp94Gly
P27SIT1.12-27.11.2007	1	97668	T	C	Rv0088-Rv0089	intergenic region		
P18SIT47.1-22.02.2008	47	141853	G	A	Rv0117	*oxyS*	oxidative stress response regulatory protein OxyS	none
P45SIT1.18-28.09.2011	1	146099	T	G	Rv0120c	*fusA2*	elongation factor G	Gln558Pro
P39SIT1.15-03.10.2019	1	158972	T	G	Rv0131c	*fadE1*	acyl-CoA dehydrogenase FadE1	none
P48SIT42.9-23.05.2019	42	159036	A	G	Rv0131c	*fadE1*	acyl-CoA dehydrogenase FadE1	Met208Thr
P45SIT1.18-28.09.2011	1	160426	G	T	Rv0132c	*fgd2*	F420-dependent glucose-6-phosphate dehydrogenase	Tyr119*
P11SIT1.5-24.09.2014	1	175159	C	T	Rv0148		short-chain type dehydrogenase/ reductase	none
P47SIT42.8-26.10.2011	42	219959	G	C	Rv0188-Rv0189c	intergenic region		
P27SIT1.12-27.11.2007	1	275791	T	G	Rv0230c	*php*	phospho-triesterase	Lys58Thr
P15SIT254.4-24.09.2014	254	299045	G	C	Rv0248c		succinate dehydrogenase flavoprotein subunit	Arg587Gly
P27SIT1.12-27.11.2007	1	469751	C	T	Rv0390		hypothetical protein	Ala54Val
P33SIT53.8-24.11.2010	53	503464	C	T	Rv0417-Rv0418	intergenic region		
P9SIT42.1-15.04.2014	42	547401	C	T	Rv0456B	*mazE1*	antitoxin MazE1	none

P34SIT190.1-05.03.2015	190	574318	T	G	Rv0485		transcriptional regulator	Ile112Ser
P6SIT53.1-23.05.2014	53	620139	C	T	Rv0529	*ccsA*	cytochrome C-type biogenesis protein CcsA	none
P27SIT1.12-27.11.2007	1	632624	C	T	Rv0540		hypothetical protein	Gln85*
P27SIT1.12-27.11.2007	1	656555	A	G	Rv0565c		monooxygenase	Tyr306His
P11SIT1.5-13.02.2013	1	699027	G	C	Rv0601c-Rv0602c	intergenic region		
P8SIT1.4-02.07.2014	1	699442	A	G	Rv0602c	*tcrA*	two component DNA binding transcriptional regulator TcrA	Tyr120His
P18SIT47.1-22.02.2008	47	814472	A	C	Rv0721	*rpsE*	30S ribosomal protein S5	Ile49Leu
P11SIT1.5-24.09.2014	1	818792	G	T	Rv0726c		S-adenosyl-methionine-dependent methyltransferase	none
P33SIT53.8-24.11.2010	53	841448	C	A	Rv0749	*vapC31*	ribonuclease VapC31	Thr74Asn
P31SIT53.6-04.11.2013	53	853366	T	G	Rv0758	*phoR*	two component system response sensor kinase PhoR	Leu324Arg
P41SIT1.16-24.05.2019	1	908095	T	G	Rv0813c-Rv0814c	intergenic region		
P18SIT47.1-17.04.2014	47	935698	T	G	Rv0839		hypothetical protein	Leu41Trp
P3SIT254.1-14.04.2014	254	948203	C	T	Rv0851c		short-chain type dehydrogenase/ reductase	Ser89Asn
P8SIT1.4-02.07.2014	1	949823	C	T	Rv0853c	*pdc*	alpha-keto-acid decarboxylase	none
P40SIT254.5-04.10.2006	254	975602	C	T	Rv0876c		transmembrane protein	none
P36SIT1.13-09.03.2011	1	1039478	A	G	Rv0931c	*pknD*	serine/ threonine-protein kinase PknD	Val146Ala
P33SIT53.8-19.06.2009	53	1045235	G	C	Rv0937c	*mku*	non-homologous end joining protein Ku	none
P40SIT254.5-27.09.2019	254	1067407	C	T	Rv0955		integral membrane protein	none
P40SIT254.5-27.09.2019	254	1067805	C	G	Rv0956	*purN*	phosphoribosyl-glycinamide formyl-transferase PurN	Thr82Ser

P17SIT156.1-01.02.2013	156	1104026	C	G	Rv0987		adhesion component ABC transporter permease	none
P9SIT42.1-15.04.2014	42	1235754	C	T	Rv1109c		hypothetical protein	none
P17SIT156.1-01.02.2013	156	1549696	G	A	Rv1376		hypothetical protein	none
P40SIT254.5-27.09.2019	254	1598915	A	G	Rv1424c		membrane protein	Leu247Ser
P44SIT254.6-28.11.2019	254	1853224	G	A	Rv1643	*rplT*	50S ribosomal protein L20	Arg14Gln
P33SIT53.8-24.11.2010	53	2123145	C	A	Rv1872c	*lldD2*	L-lactate dehydrogenase	Val3Phe
P9SIT42.1-15.10.2003	42	2192178	T	C	Rv1939		oxidoreductase	Cys29Arg
P33SIT53.8-24.11.2010	53	2197910	A	G	Rv1946c	*lppG*	lipoprotein	none
P11SIT1.5-24.09.2014	1	2202120	G	C	Rv1955-Rv1956	intergenic region		
P34SIT190.1-05.03.2015	190	2211066	C	A	Rv1967	*mce3B*	Mce family protein Mce3B	Gln156Lys
P6SIT53.1-23.05.2014	53	2254827	G	A	Rv2006	*otsB1*	trehalose-6-phosphate phosphatase OtsB	none
P17SIT156.1-01.02.2013	156	2273246	G	A	Rv2027c	*dosT*	two component sensor histidine kinase DosT	none
P44SIT254.6-13.02.2008	254	2308386	G	A	Rv2051c	*ppm1*	polyprenol-monophospho-mannose synthase	none
P17SIT156.1-01.07.2014	156	2321704	C	A	Rv2064	*cobG*	precorrin-3B synthase	Ser85*
P15SIT254.4-24.09.2014	254	2352637	A	C	Rv2093c	*tatC*	Sec-independent protein translocase transmembrane protein TatC	none
P44SIT254.6-28.11.2019	254	2361222	A	C	Rv2101	*helZ*	helicase HelZ	Glu328Ala
P45SIT1.18-28.09.2011	1	2429652	T	C	Rv2166c		transcriptional regulator MraZ	none
P11SIT1.5-24.09.2014	1	2480450	G	A	Rv2214c	*ephD*	oxidoreductase EphD	none
P41SIT1.16-24.05.2019	1	2570881	C	A	Rv2299c	*htpG*	chaperone protein HtpG	none
P19SIT42.3-24.07.2014	42	2680992	G	T	Rv2387		hypothetical protein	none

P8SIT1.4-02.07.2014	1	2778624	G	A	Rv2476c	*gdh*	NAD-dependent glutamate dehydrogenase	none
P27SIT1.12-27.11.2007	1	2959723	A	G	Rv2633c		hypothetical protein	Val33Ala
P23SIT1.8-02.04.2009	1	3065994	A	G	Rv2752c		ribonuclease J	none
P11SIT1.5-13.02.2013	1	3176848	T	C	Rv2864c		penicillin-binding lipoprotein	Thr140Ala
P9SIT42.1-15.04.2014	42	3354700	G	A	Rv2996c	*serA1*	D-3-phospho-glycerate dehydrogenase	none
P15SIT254.4-24.09.2014	254	3421869	A	G	Rv3060c		GntR family transcriptional regulator	Trp449Arg
P17SIT156.1-01.02.2013	156	3514327	G	A	Rv3148	*nuoD*	NADH-quinone oxidoreductase subunit D	none
P32SIT53.7-15.01.2008	53	3597695	G	C	Rv3221c	*TB7.3*	acetyl-CoA carboxylase biotin carboxyl carrier protein subunit	Ile24Met
P21SIT1.7-10.03.2014	1	3597849	A	C	Rv3221c-Rv3221A	intergenic region		
P45SIT1.18-28.09.2011	1	3629316	T	C	Rv3248c	*sahH*	Adenosyl-homo-cysteinase	His111Arg
P18SIT47.1-17.04.2014	47	3645052	A	G	Rv3264c	*manB*	D-alpha-D-mannose-1-phosphate guanylyl-transferase ManB	Phe309Ser
P15SIT254.4-24.09.2014	254	3781510	G	A	Rv3370c	*dnaE2*	error-prone DNA polymerase	none
P11SIT1.5-24.09.2014	1	3786508	G	A	Rv3372	*otsB2*	trehalose 6-phosphate phosphatase	none
P19SIT42.3-24.07.2014	42	4010008	G	A	Rv3568c	*hsaC*	extradiol dioxygenase	none
P23SIT1.8-21.06.2011	1	4049151	T	C	Rv3608c	*folP1*	dihydro-pteroate synthase	Glu277Gly
P3SIT254.1-14.04.2014	254	4101952	G	C	Rv3662c		hypothetical protein	none
P27SIT1.12-27.11.2007	1	4145585	T	C	Rv3702c	*egtC*	amido-hydrolase EgtC	none
P22SIT42.4-25.10.2013	42	4308372	G	A	Rv3834c	*serS*	serine--tRNA ligase	none
P40SIT254.5-04.10.2006	254	4352260	G	C	Rv3873-Rv3874	intergenic region		
P40SIT254.5-27.09.2019	254	4360108	G	A	Rv3879c-Rv3880c	intergenic region		

P17SIT156.1-01.02.2013	156	4385473	C	T	Rv3900c		hypothetical protein	Gly279Asp
